# RETRACTION: FLOT1 promotes tumor development, induces epithelial–mesenchymal transition, and modulates the cell cycle by regulating the Erk/Akt signaling pathway in lung adenocarcinoma

**DOI:** 10.1111/1759-7714.70326

**Published:** 2026-06-17

## Abstract

**RETRACTION**: ZhangL. , MaoY. , MaoQ. , FanW. , XuL. , ChenY. , XuL. and WangJ. , “FLOT1 promotes tumor development, induces epithelial–mesenchymal transition, and modulates the cell cycle by regulating the Erk/Akt signaling pathway in lung adenocarcinoma,” Thoracic Cancer 10, no. 4 (2019): 909‐917, 10.1111/1759-7714.13027.30838797 PMC6449277

The above article, published online on 05 March 2019 in Wiley Online Library (wileyonlinelibrary.com), and its correction, (https://doi.org/10.1111/1759‐7714.14990), has been retracted by agreement between the journal Editor‐in‐Chief, Tateaki Naito; and John Wiley & Sons Australia, Ltd. The retraction has been agreed upon following concerns raised by a third party. An investigation identified duplication between the OEFLOT1_NC flow cytometry plot presented in Figure 2f of this article and a plot published previously by a different author group. The authors were contacted for comment and supporting data, but did not respond. In light of these findings, the editors consider the results and conclusions to be unreliable. The authors did not respond to our notice of retraction.

**RETRACTION**: L. Zhang , Y. Mao , Q. Mao , W. Fan , L. Xu , Y. Chen , L. Xu and J. Wang , “FLOT1 promotes tumor development, induces epithelial–mesenchymal transition, and modulates the cell cycle by regulating the Erk/Akt signaling pathway in lung adenocarcinoma,” Thoracic Cancer 10, no. 4 (2019): 909‐917, 10.1111/1759-7714.13027.30838797 PMC6449277

The above article, published online on 05 March 2019 in Wiley Online Library (wileyonlinelibrary.com), and its correction, (https://doi.org/10.1111/1759‐7714.14990), has been retracted by agreement between the journal Editor‐in‐Chief, Tateaki Naito; and John Wiley & Sons Australia, Ltd. The retraction has been agreed upon following concerns raised by a third party. An investigation identified duplication between the OEFLOT1_NC flow cytometry plot presented in Figure 2f of this article and a plot published previously by a different author group. The authors were contacted for comment and supporting data, but did not respond. In light of these findings, the editors consider the results and conclusions to be unreliable. The authors did not respond to our notice of retraction.

